# Mutations in the Hemagglutinin Stalk Domain Do Not Permit Escape from a Protective, Stalk-Based Vaccine-Induced Immune Response in the Mouse Model

**DOI:** 10.1128/mBio.03617-20

**Published:** 2021-02-16

**Authors:** Ericka Kirkpatrick Roubidoux, Juan Manuel Carreño, Meagan McMahon, Kaijun Jiang, Harm van Bakel, Patrick Wilson, Florian Krammer

**Affiliations:** a Department of Microbiology, Icahn School of Medicine at Mount Sinai, New York, New York, USA; b Graduate School of Biomedical Sciences, Icahn School of Medicine at Mount Sinai, New York, New York, USA; c Department of Genetics and Genomic Sciences, Icahn School of Medicine at Mount Sinai, New York, New York, USA; d Icahn Institute for Data Science and Genomic Technology, Icahn School of Medicine at Mount Sinai, New York, New York, USA; e Department of Medicine, Section of Rheumatology, the Knapp Center for Lupus and Immunology, University of Chicago, Chicago, Illinois, USA; St. Jude Children’s Research Hospital

**Keywords:** influenza, stalk-based vaccines, universal influenza virus vaccines

## Abstract

Current seasonal influenza virus vaccines target regions of the hemagglutinin (HA) head domain that undergo constant antigenic change, forcing the painstaking annual reformulation of vaccines. The development of broadly protective or universal influenza virus vaccines that induce cross-reactive, protective immune responses could circumvent the need to reformulate current seasonal vaccines. Many of these vaccine candidates target the HA stalk domain, which displays epitopes conserved within and across influenza virus subtypes, including those with pandemic potential. While HA head-mediated antigenic drift is well understood, the potential for antigenic drift in the stalk domain is understudied. Using a panel of HA stalk-specific monoclonal antibodies (MAbs), we applied selection pressure to the stalk domain of A/Netherlands/602/2009 (pdmH1N1) to determine fitness and phenotypes of escape mutant viruses (EMVs). We found that HA stalk MAbs with lower cross-reactivity caused single HA stalk escape mutations, whereas MAbs with broader cross-reactivity forced multiple mutations in the HA. Each escape mutant virus greatly decreased mAb neutralizing activity, but escape mutations did not always ablate MAb binding or Fc-Fc receptor-based effector functions. Escape mutant viruses were not attenuated *in vitro* but showed attenuation in an *in vivo* mouse model. Importantly, mice vaccinated with a chimeric HA universal vaccine candidate were protected from lethal challenge with EMVs despite these challenge viruses containing escape mutations in the stalk domain. Our study indicates that while the HA stalk domain can mutate under strong MAb selection pressure, mutant viruses may have attenuated phenotypes and do not evade a polyclonal, stalk-based vaccine-induced response.

## INTRODUCTION

Influenza viruses are highly contagious respiratory pathogens that affect a large proportion of the world’s population. The morbidity and mortality caused by influenza virus infections has led to a massive effort to track and control infections worldwide. Since 1942, annual influenza virus vaccines have been utilized to try to mitigate influenza virus infections (1). Different types of vaccines have been produced, including monovalent, trivalent, or quadrivalent vaccines containing live attenuated virus, inactivated whole virus, or inactivated split virus. Recent vaccine formulations have also incorporated recombinant protein as vaccine antigens (2, 3). Despite improvements in immunogenicity and production over the years, influenza virus vaccines generally have low effectiveness each season (4–7). Low vaccine effectiveness stems from the ability of influenza virus, like many RNA viruses, to mutate rapidly (8). Mutations acquired in segments that encode the virus’ major glycoproteins, hemagglutinin (HA) and neuraminidase (NA), are drivers of antigenic changes that can lead to escape from preexisting immune responses. This process is known as antigenic drift and causes a need for annual reformulation of current seasonal influenza virus vaccines (2, 3).

Generally, protective immune responses are mediated by neutralizing antibodies that target the HA head domain. These antibodies block virus receptor binding site and prevent initial infection (9, 10). The major antigenic sites of the HA head domain are highly plastic, evolving regions of the protein (11–15). Targeting these sites allows for a protective, yet narrow, antibody response. To further broaden protective antibody responses, efforts have focused on evaluating the protective potential of conserved sites of the HA protein, such as the stalk domain. This domain is relatively conserved within and across IAV group 1, IAV group 2, and influenza B virus HAs (16–20). Several universal influenza virus vaccine candidates that induce long-lasting and broad protection are under development. These candidates are designed to redirect the immune response from the head domain toward the stalk domain to induce cross-reactive antibody responses. Among the stalk-based universal influenza vaccine candidates is the chimeric HA (cHA) approach (21–25). cHA vaccines are designed to have a single, conserved stalk domain combined with an “exotic” avian HA head domain such as H5, H8, H11, or H13. A prime-boost-boost vaccination strategy with different cHA constructs increased the levels of stalk-reactive antibodies in preclinical ferret models (26) and in a phase 1 clinical trial (27).

However, it is unclear whether sufficient immune pressure from stalk-based vaccines could induce antigenic drift in this conserved region. Over the years, cross-reactive, stalk-specific antibodies have been isolated from infected or immunized individuals or mice (28–32). We used six previously described monoclonal antibodies (MAbs) that target the HA stalk domain as tools for the generation of HA stalk escape mutant viruses (EMVs). EMVs were generated using A/Netherlands/602/2009 (pdmH1N1) passaged with increasing amounts of MAb to mimic the immune selection pressure that would be present after stalk-based vaccinations. We were able to generate EMVs using these MAbs and evaluated the impact of stalk escape mutations on cross-reactive MAb neutralization, binding, Fc-Fc receptor-based effector functions, and impact on *in vitro* and *in vivo* fitness. Our data support the growing consensus that stalk-based immunity is diverse enough to provide protection against viruses containing mutations in the HA stalk domain.

## RESULTS

### Generation of HA stalk escape mutant viruses using a panel of cross-reactive stalk-specific MAbs.

**FIG 1:**
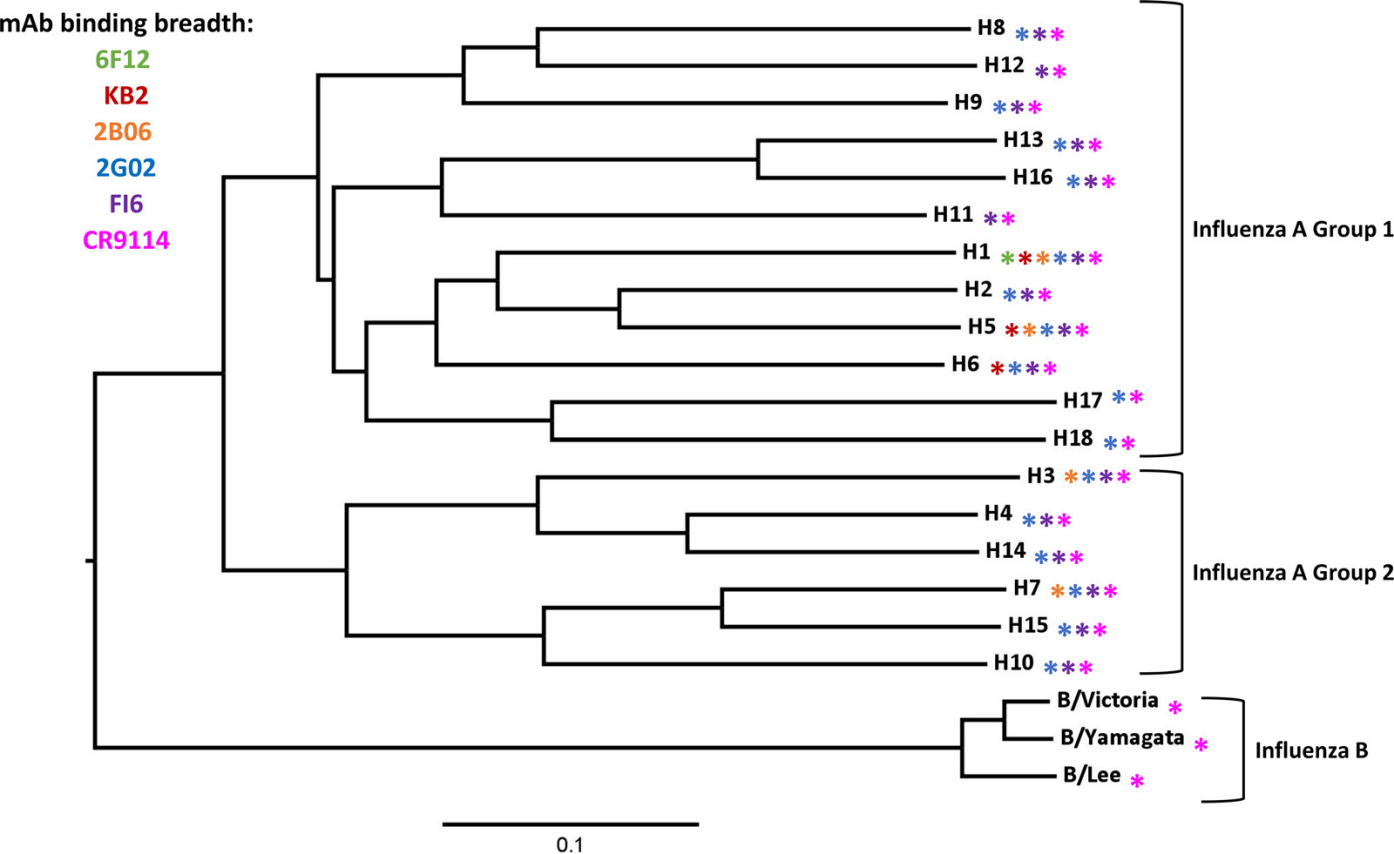
Stalk specific MAbs have various degrees of breadth. The phylogenetic tree shows representative HA protein sequences for each subtype. Influenza A group 1 HA, influenza A group 2 HA, and influenza B viruses are indicated by brackets. HA subtypes that are recognized by a given MAb are indicated by colored stars. 6F12 only binds HAs within group 1 and is shown in green. KB2 binds H1, H5 and H6 and is shown in red. 2B06 binds H1, H5, H3, and H7 and is shown in orange. 2G02, FI6, and CR9114 MAbs are highly cross-reactive, and their breadth is illustrated by blue, purple, or magenta, respectively. The scale bar represents a 10% difference in amino acid sequence.

To better understand the effects of escape mutations on the HA stalk domain, we used a panel of stalk-specific MAbs: 6F12 (30), KB2 (32), 045-051310-2B06 (2B06) (33), 05-2G02 (2G02) (34), FI6v3 (FI6) (29), and CR9114 (31). These MAbs were selected for their cross-reactive binding (as depicted in Fig. 1), their ability to neutralize virus *in vitro* and the ability to protect against lethal challenge *in vivo.* 6F12, a mouse MAb, has the lowest breadth of cross-reactivity since it can only cross-react within the H1 subtype (30). We used an additional mouse MAb, KB2, which cross-reacts with both H1, H5, and H6 (32). We also selected a panel of four human cross-reactive anti-stalk MAbs. MAb 2B06 has demonstrated binding to group 1 H1 and H5 and group 2 H3 and H7 HAs, although binding to other subtypes has not been thoroughly investigated (33). MAb 2G02 has broader cross-reactivity between groups 1 and 2 (33, 34). MAbs FI6 and CR9114 cross-react with all subtypes of IAV, with CR9114 also interacting with IBVs (29, 31).

**FIG 2:**
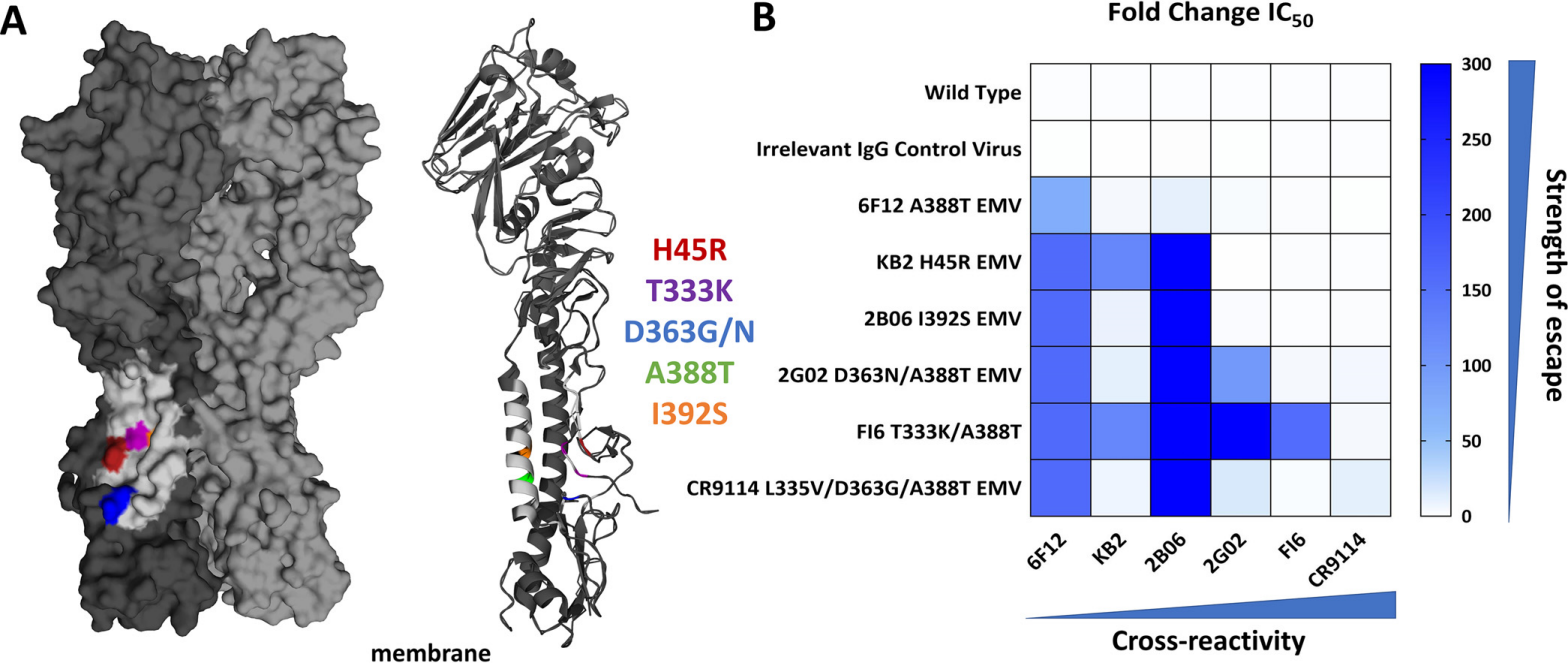
HA stalk mutations led to escape from MAb neutralization. (A) Location of escape mutations on the HA of A/Puerto Rico/8/1934 HA (PDB ID 1RU7 [54]). A three-dimensional (3D) version of the HA trimer is shown on the left. On the trimer, one monomer is modeled in dark gray and identifies the acquired HA stalk mutations. The region in light gray on this monomer is a summary of previously reported stalk MAb epitopes (11). A ribbon structure of a single HA monomer is shown on the right. H45R is indicated in red, T333K is indicated in purple, D363G/N is indicated in blue, and I392S is indicated in orange. The additional mutation A388T is not visible on the 3D HA structure but is visible on the HA2 α helix of the ribbon structure. (B) Heat map of the fold change in the 50% inhibitory concentration (IC_50_) for each EMV compared to wild-type A/Netherlands/602/2009. Higher fold changes indicate stronger escape phenotypes. MAbs are ordered by increasing breadth, which is indicated along the bottom of the figure.

EMVs were generated using a pdmH1N1 virus that was copassaged with increasing amounts of stalk MAbs using gradual selection pressure in Madin-Darby canine kidney cells (MDCKs). We began with 0.25× the 50% inhibitory concentration (IC_50_) of MAb mixed with virus at a 0.01 multiplicity of infection (MOI) and continued passaging 10 times to 128× IC_50_ (doubling the IC_50_ at every passage), with the exception of CR9114, which was passaged 15 times. The highly cross-reactive nature of CR9114 may be why it was especially difficult to generate this EMV. As a control, we also passaged pdmH1N1 with an irrelevant IgG control MAb 1C12 (anti-ebolavirus glycoprotein [35]) up to passage 6. Virus was plaque purified from the first passage that was able to grow in the presence of 128× IC_50_ of MAb (which was tested at every passage). The first passages where an escape phenotype was observed, along with acquired mutations, are listed in Table 1. MAb6F12 escape was mediated by a single stalk mutation of A388T (numbering from methionine). The mutation is located on the inside of the α-helix of HA2 close to other known stalk MAb epitopes. MAb KB2 caused a single stalk escape mutation of H45R (Fig. 2A). An I392S substitution led to the 2B06 escape EMV (Fig. 2A), while the remaining three MAbs needed multiple, yet shared, stalk mutations for their escape phenotype. MAb 2G02 led to the individual mutation D363N and a shared A388T mutation. The FI6 EMV had an individual T333K mutation and shared A388T with the other EMVs. Finally, CR9114 escape led to three stalk mutations: a unique mutation of L335V, D363G (shared residue position with the 2G02 EMV but a different substitution), and the A388T mutation (Fig. 2A). All viruses, including the irrelevant IgG control virus, acquired three cell culture adaptations in the HA head domain: R62K, D239G, and R240Q. To confirm that our mutations are similar to those previously identified, we compared escape mutations from each MAb in our study with critical residues indicated in literature. 6F12 caused a HA2 A44V mutation, which matches to the A388 residue, although we observed an A388T substitution (30). The critical residue for KB2 has been previously reported as H45R (36). The residues T333 and D363 were also previously reported to be critical for FI6 and CR9114, respectively (29, 31). Although L335 and I392 were not specifically listed in the literature, interacting residues for both CR9114 and 2B06 are near these sites at I362 and I389. These comparisons allowed us to infer that the observed stalk mutations in our study were likely the drivers of our HA EMV phenotypes.

**TABLE 1:** Mutations found in EMVs compared to wild-type A/Netherlands/602/2009

Isolate	Passage	Mutation(s)^*a*^				
		PB2	PA	HA	NA	NS1
Irrelevant IgG control virus	Arbitrarily stopped at passage 6		V407I	R62K, K163N, D239G, R240Q	G454D	G179R, I198L
6F12 A388T EMV	8			R62K, D239G, R240Q, **A388T**		
KB2 H45R EMV	9			**H45R**, R62K, **A152S**, D239G, R240Q	V53I	
2B06 I392S EMV	9	D195N	F35L	R62K, D239G, R240Q, **I392S**		
2G02 D363N/A388T EMV	9			R62K, D239G, R240Q, **D363N**, **A388T**	I54N	
FI6 T333K/A388T EMV	8		V100L	R62K, D239G, R240Q **T333K**, **A388T**		
CR9114 L335V/D363G/A388T EMV	15	ND	ND	R62K, D239G, R240Q, **L335V**, **D363G**, **A388T**	ND	ND

^a^ Only segments with mutations are listed. All numbering is from methionine. ND, segments that have not been sequenced. Boldfaced HA mutations are not found in the irrelevant IgG control virus.

### A majority of escape mutations appear simultaneously and are stable *in vivo*.

To assess whether escape mutations occurred concurrently or appeared individually over time, we examined the consensus sequences of RNA extracted from cell culture supernatants at each passage. For all EMVs, cell culture adaptations appeared around passage 2. As expected, escape mutations began to appear in the last one to two passages. The exception is the CR9114 EMV, where L335V appeared at passage 9, D363G at passage 11, and A388T at passage 12. For some EMVs, the mutation appeared before the EMV was identified and plaque purified. This may be because the MAb neutralized a majority of the viral population so that the screen was not sensitive enough to detect EMVs at low frequency. We additionally assessed whether a passage through mice would cause EMVs to revert back to wild-type sequences *in vivo*. We found that escape mutations were maintained in virus from mouse lung homogenates for a majority of the EMVs. For instance, cell culture adaptations did not change. However, the 6F12 A388T EMV escape mutation was only present in one of three replicates. We did not identify any changes in the HA genes of the KB2 H45R, 2B06 I392S, 2G02 D363N/A388T, and FI6 T333K/A388T EMVs. The CR9114 L335V/D363G/A388T EMV had some changes after mouse passaging. It lost the L335V mutation and gained a new mutation, N394S, in all three replicates. In addition, one of three viruses contained a D363N mutation instead of D363G. Overall, these data suggest that once acquired, escape mutations may continue to persist in circulating viruses.

### Stalk MAb escape mutant viruses evade neutralization from their specific MAb along with MAbs with lower cross-reactive breadth.

**FIG 3:**
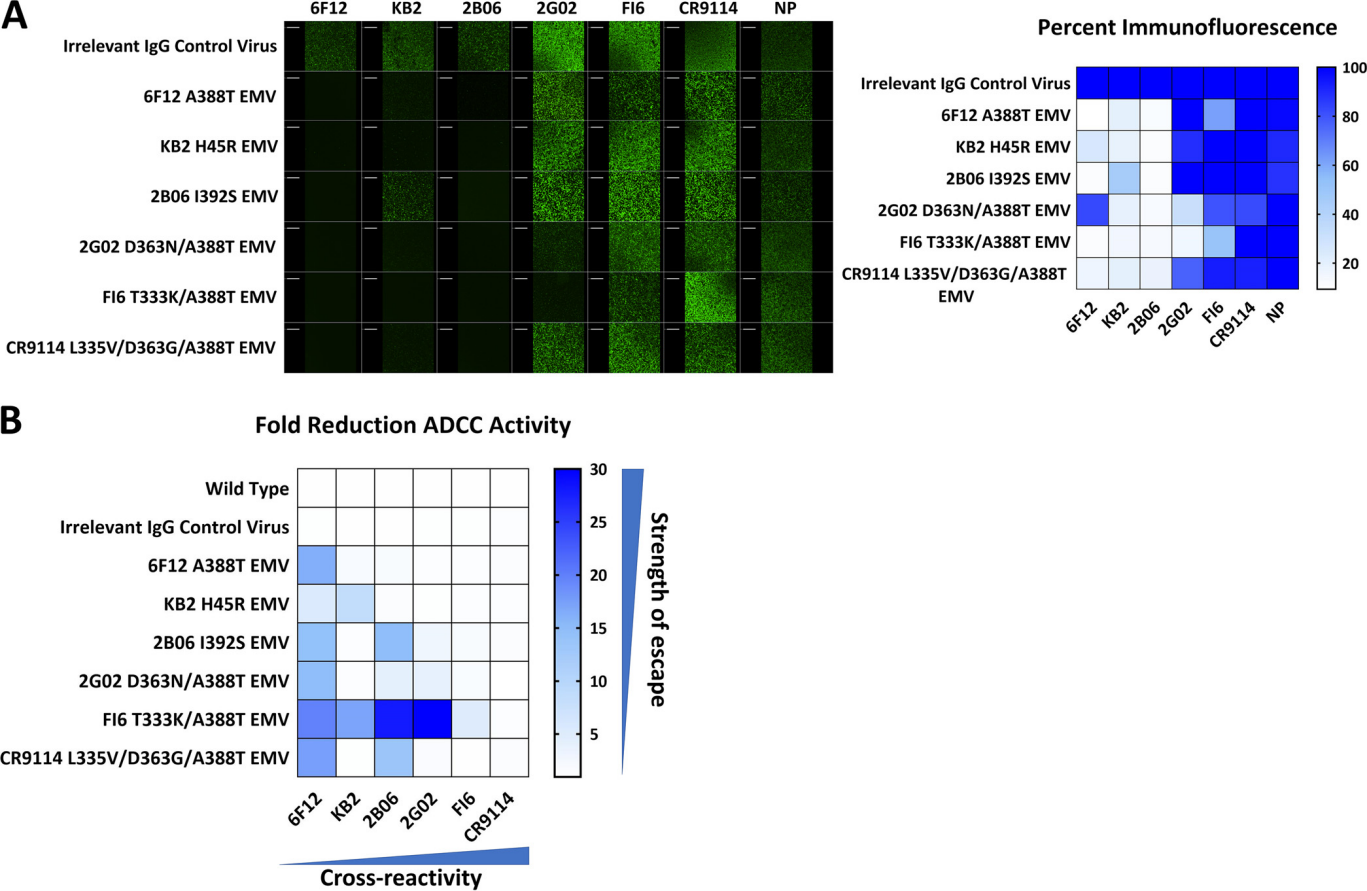
Escape from MAb binding correlates with a reduction of ADCC activity. (A) Immunofluorescence assay of MDCK cells infected with EMVs. MAbs were added at 30 μg/well and are indicated at the top of the figure. On the right, a heatmap illustrates the percent fluorescence for each image. (B) Heat map of the fold reduction of ADCC activity of each EMV against the panel of MAbs. The fold reduction was determined by dividing the total AUC value of the wild-type virus by the AUC for a given EMV. A higher fold reduction means a stronger escape phenotype.

To determine whether a stalk mutation could confer escape to more than one MAb, we used plaque reduction assays to determine the IC_50_ value of each MAb against all EMVs and then calculated the fold increase compared to wild-type pdmH1N1 (Table 2) (Fig. 2B). We observed no significant IC_50_ changes between the irrelevant IgG control virus and the wild-type virus. The EMV of the MAb with the lowest breadth, 6F12 A388T EMV, had a 73-fold change in IC_50_ against 6F12, a 4.5-fold change against KB2 and a 11-fold change against 2B06 (Fig. 2B). The H45R escape mutation from KB2 caused a 159-fold change in IC_50_ against 6F12, 122-fold against KB2 and 298-fold against 2B06 (Fig. 2B). The human MAb 2B06 generated the I392S EMV that had a 159-fold change in IC_50_ against 6F12, 9-fold change against KB2, and a 298-fold change against 2B06 (Fig. 2B). The broader human MAbs 2G02, FI6 and CR9114 generated EMVs that caused large fold changes in IC_50_ of their respective MAbs and also caused changes in all MAbs with lower breadth. For instance, the 2G02 D363N/A388T EMV had a 159-fold change against 6F12, an 11-fold change against KB2, a 298-fold change against 2B06, and a 99-fold change against 2G02 (Fig. 2B). The FI6 T333K/A388T EMV had a 159-fold change against 6F12, 122-fold change against KB2, 298-fold change against 2B06, 416-fold change against 2G02, and a 157-fold change against FI6 (Fig. 3). The final EMV, CR9114 L335V/D363G/A388T, had changes in IC_50_s against all of the MAbs: 159-fold for 6F12, 7-fold for KB2, 298-fold for 2B06, 17-fold for FI6, and 11.5-fold for CR9114 (Fig. 2A). While some of these fold changes may not seem significant (i.e., the 11.5-fold change for the CR9114 L335V/D363G/A388T mutation versus CR9114), all changes indicate increases from nanomolar to micromolar IC_50_ values. These observations suggest that there is a balance between ease of escape from each MAb and the total effects of each set of escape mutations.

**TABLE 2:** IC_50_ values for the MAb panel against EMVs

Isolate	MAb IC_50_ (μg/ml)						
	7B2	6F12	KB2	2B06	2G02	FI6	CR9114
Wild type	0.0047	0.6274	0.8184	0.3351	0.2404	0.1178	0.1028
Irrelevant IgG control virus	0.0021	0.3639	0.255	0.2838	0.1277	0.1084	0.1035
6F12 A388T EMV	0.0011	>100	>100	>100	0.0965	0.1912	0.108
KB2 H45R EMV	0.0032	46.02	3.643	3.687	0.7043	0.2094	0.0626
2B06 I392S EMV	0.0032	>100	7.456	>100	0.6204	0.1773	0.1679
2G02 D363N/A388T EMV	0.0032	>100	9.362	>100	23.76	0.5036	0.5509
FI6 T333K/A388T EMV	0.0032	>100	>100	>100	>100	18.55	0.455
CR9114 L335V/D363G/A388T EMV	0.0032	>100	6.1	>100	4.076	0.2735	1.192

### Stalk MAbs with the highest breadth retain binding and Fc-FcR effector functions toward escape mutant viruses.

While it has been observed that HA head escape mutations lead to both escape from neutralization and MAb binding, it has also been shown that escape from stalk specific MAbs does not always have this phenotype (33). We investigated whether our stalk EMVs escaped binding from any MAbs in the panel using an immunofluorescence assay. All EMVs had a loss or large reduction in the binding activity of 6F12, KB2, and 2B06 (Fig. 3A). In addition, the 2G02 D363N/A388T and FI6 T333K/A388T EMVs reduced binding of 2G02. The CR9114 L335V/D363G/A388T EMV was still bound by CR9114, along with 2G02 and FI6 (Fig. 3A). Binding of the broadest cross-reactive MAbs, FI6 and CR9114, was maintained for all EMVs.

Several of the stalk-specific MAbs in our panel have been previously reported to have Fc-FcR based effector functions that can mediate protection *in vivo* (37–40). We noticed that escape from neutralization was not always matched with an escape from binding, which led to a curiosity to determine whether either escape from neutralization or binding was more indicative of changes in MAb Fc-effector functions. To measure effector functions, we used the Promega antibody-dependent cell-mediated cytotoxicity (ADCC) reporter assay kit with either mFcγRIV (mouse) or FcγRIIIa (human) effector cells (41). ADCC activity was quantified by calculating the area under the curve (AUC) for each virus-MAb combination. We calculated the fold reduction by comparing these values to the AUC values measured with wild-type virus. All stalk MAbs had ADCC activity against the wild type and irrelevant IgG control viruses (Fig. 3B). The 6F12 A388T EMV only caused a reduction of ADCC activity against 6F12, but all other MAbs still showed activity to this EMV similar to the wild-type virus (Fig. 3B). The KB2 H45R EMV had a reduction of ADCC activity with both 6F12 and KB2 (Fig. 3B). The MAbs FI6 and CR9114 retained their activity against all EMVs (Fig. 3B). We observed correlations between all three measurements of escape phenotypes. To quantify changes in binding, we calculated the percent fluorescence by comparing the EMV raw fluorescence intensity to that of the wild-type virus. MAbs that escaped neutralization also escaped binding and ADCC activity, with *R* values of −0.6326 (correlation between %binding and fold change in IC_50_) and 0.7457 (correlation between fold change in IC_50_ and fold change in ADCC activity), respectively. However, MAbs that retained binding also had the smallest reductions in ADCC activity, with an *R* of −0.5413 (correlation between %binding and fold change in ADCC activity). These data suggest that while stalk EMVs may no longer be neutralized by many MAbs, they may still be controlled by stalk-specific MAbs through their retained ADCC activity.

### Escape mutant viruses show no fitness loss *in vitro* but are attenuated *in vivo*.

**FIG 4:**
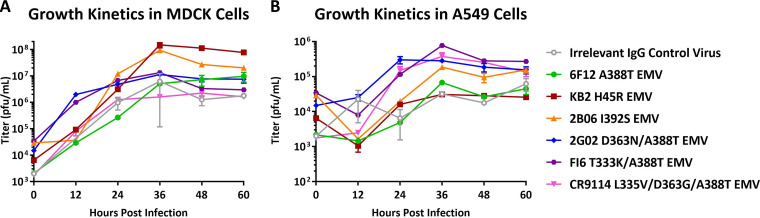
Growth kinetics of EMVs in cell culture. (A) Growth kinetics of EMVs in MDCK cells. (B) Growth kinetics of EMVs in A549 cells. Each time point had two biological replicates.

It is important to evaluate the fitness of EMVs to understand the impact of each set of HA stalk mutations. To measure fitness *in vitro*, we conducted growth curve experiments using MDCK and A549 (human lung epithelial) cells (Fig. 4). Cells were infected with an MOI of 0.01 and supernatant was collected every 12 h until 60 h postinfection. Virus titers were quantified using plaque assays on MDCK cells. Virus titers were higher in MDCK cells, but no virus showed obvious attenuated or increased growth compared to the irrelevant IgG control virus (Fig. 4). These results are not surprising considering that each stalk EMV was generated by serial passage on MDCK cells and that they may have adapted to cell culture.

Next, to determine the EMV’s fitness *in vivo*, we intranasally infected BALB/c mice with dilutions of virus in order to calculate the 50% murine lethal dose (mLD_50_). Our irrelevant IgG control virus was highly lethal, with an mLD_50_ of 7 PFU/mouse (Table 3). Each EMV was attenuated compared to the irrelevant IgG control virus. 6F12 A388T was attenuated at an mLD_50_ of 5.62 × 10^3^ PFU/mouse (Table 3). The KB2 H45R EMV was the least attenuated with an ∼10-fold increase in mLD_50_ compared to the irrelevant IgG control virus, at 56 PFU/mouse (Table 3). The 2B06 I392S EMV was attenuated to an mLD_50_ of 3.16 × 10^3^ (Table 3). The final three EMVs—2G02 D363N/A388T, FI6 T333K/A388T, and CR9114 L335V/D363/NA388T—all had an mLD_50_ that was >10^5^ PFU/mouse (the highest dose administered) (Table 3). We therefore found that EMVs from the broadest MAbs were not lethal, and other EMVs showed various amounts of attenuation.

**TABLE 3:** Summary of EMV mLD_50_ values

Virus	mLD_50_ (PFU/mouse)
A/Netherlands/602/2009	1
Irrelevant IgG control virus	7
6F12 A388T EMV	5.62 × 10^3^
KB2 H45R EMV	56
2B06 I392S EMV	3.16 × 10^3^
2G02 D363N/A388T EMV	>10^5^
FI6 T333K/A388T EMV	>10^5^
CR9114 L335V/D363G/A388T	>10^5^

### Chimeric HA vaccination induces immune responses that remain protective against escape mutant viruses.

**FIG 5:**
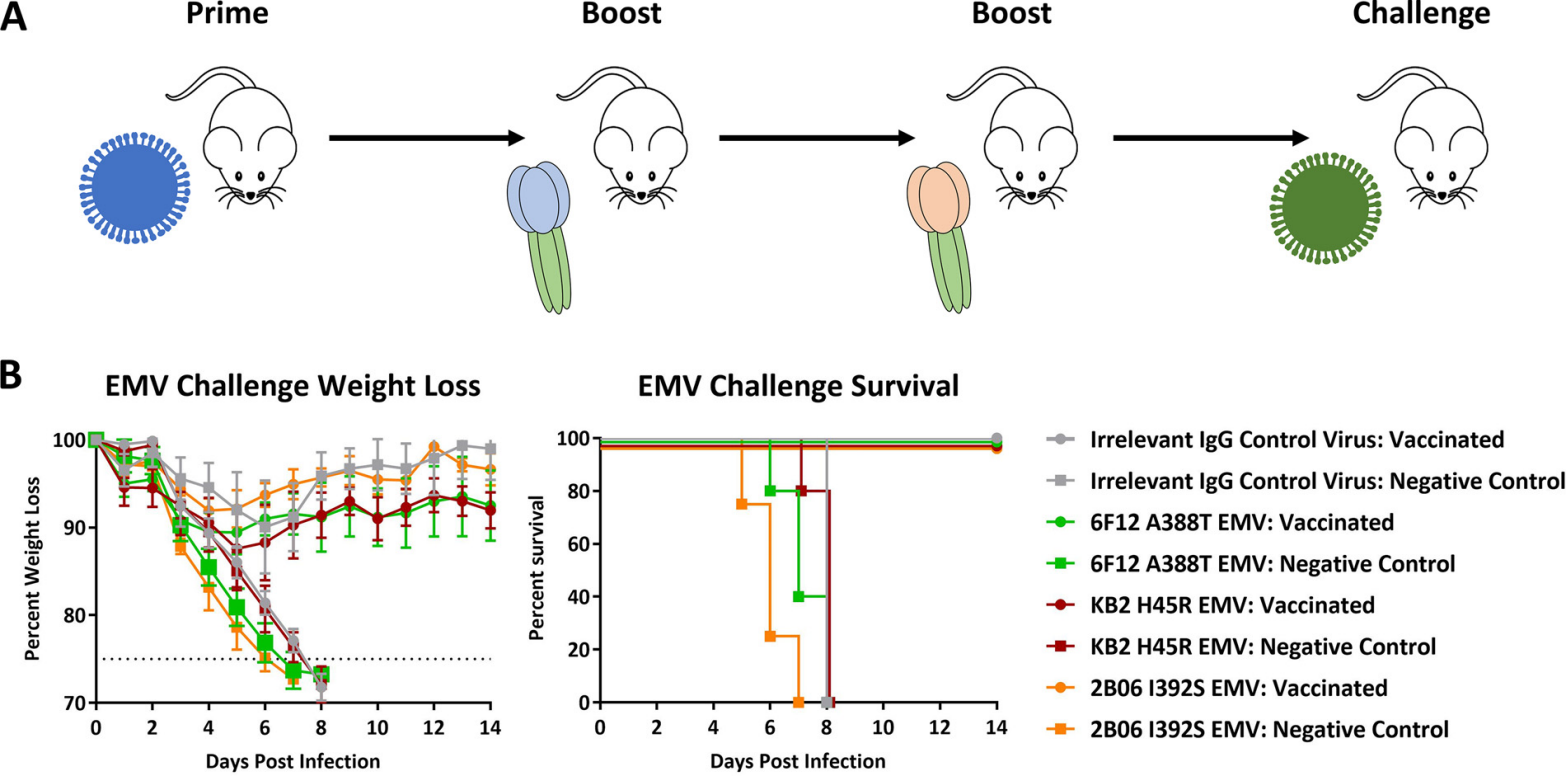
Chimeric HA vaccination protects from lethal challenge with EMVs. (A) Schematic representation of the cHA vaccination strategy. (B) Mice vaccinated using the cHA strategy described were challenged with 20× mLD_50_ of each EMV. Weight loss and survival data for vaccinated and negative-control mice are shown for the irrelevant IgG control virus, along with 6F12 A388T, KB2 H45R, and 2B06 I392S EMVs.

The escape mutations described in the present study are rarely observed in nature. In fact, we found that H45, T333, L335, D363, and A388 were 100% conserved in a data set that contained 2,905 sequences collected from 1918 to 2018 (see Fig. S1 in the supplemental material). The I392S mutation was observed in a single virus from this data set, A/Singapore/GP3441/2009, suggesting a conservation of the site of >99.9%. The highly conserved properties of the HA stalk domain make it an interesting vaccine antigen. Recently, chimeric HAs (cHAs) with a conserved pdmH1N1 stalk domain have been tested as vaccine candidates in a preclinical ferret model and in a phase 1 clinical trial (26, 27). To evaluate whether stalk escape mutations can reduce the effectiveness of this vaccination strategy, we conducted challenge experiments in cHA-vaccinated BALB/c mice. Vaccinated mice were given a prime of cH9/1 in a B/Yamagata/16/1988 viral backbone to mimic human, stalk-specific preexisting immunity (22, 26). After 3 weeks, the mice were boosted with 10 μg of poly(I·C)-adjuvanted cH8/1 protein. After an additional 3 weeks, the mice were boosted again, with poly(I·C)-adjuvanted cH5/1 protein. Mice in the negative-control group were first primed with wild-type B/Yamagata/16/1988 and boosted twice with 10 μg of poly(I·C)-adjuvanted bovine serum albumin (BSA). By 4 weeks after the final boost, all mice were challenged with 20× mLD_50_ of the irrelevant IgG control virus, 6F12 A388T EMV, KB2 H45R EMV, or 2B06 I392S EMV (Fig. 5A). All vaccinated mice were protected from lethal challenge of all EMVs. Vaccinated mice challenged with the irrelevant IgG control virus, 6F12 A388T EMV, or KB2 H45R EMV lost approximately 10% of their body weights before recovering from the infection. Vaccinated mice challenged with 2B06 I392S EMV lost barely any body weight during the course of the experiment. All negative-control mice succumbed to infection (Fig. 5B). These results demonstrate that the cHA vaccination approach induces a polyclonal antibody response that protects against viruses with changes in the HA stalk domain.

10.1128/mBio.03617-20.1 FIG S1 Shannon entropy scores for each amino acid residue of the H1 HA. Scores were calculated using the Shannon Entropy-One webserver (https://www.hiv.lanl.gov/). Higher entropy scores indicate more variability at a specific residue. Residues that were mutated in EMVs (45, 333, 335, 363, 388, and 392) are indicated by red arrows. Download FIG S1, TIF file, 2.7 MB. Copyright © 2021 Roubidoux et al. 2021 Roubidoux et al. https://creativecommons.org/licenses/by/4.0/ This content is distributed under the terms of the Creative Commons Attribution 4.0 International license.

## DISCUSSION

In this study, we used a panel of broadly reactive MAbs to understand the impact of H1 stalk escape mutations on MAb neutralization, binding, Fc-effector functions, and virus fitness. The clinical development of stalk-based vaccines is moving forward and already delivering promising results (42). However, we do not know what will happen once more pressure is applied to the stalk domain. Will there be effective antigenic drift? Here, we show that we can drive escape from neutralization by stalk-reactive antibodies, but this escape is often not complete and comes with significant fitness losses of the virus *in vivo*. This is the first time that this has been demonstrated in detail. EMVs showed a pattern where they were able to escape MAbs with lower breadth than their generating MAbs; however, MAbs with higher breadth remained neutralizing. For example, 6F12 A388T EMV escaped from 6F12, but the other MAbs in the panel remained neutralizing. The FI6 T333K/A388T EMV escaped neutralization from all MAbs except for the broadest, CR9114. Some MAbs retained binding to EMVs despite escape from neutralization. The broadest MAbs FI6 and CR9114 retained binding to all viruses, including their EMVs. We used total fluorescence of each well in the immunofluorescence assay to calculate percent binding compared to the wild-type pdmH1N1. The percent reduction in fluorescence correlated with the fold reduction of ADCC activity. This suggests that ADCC reporter activity depends on binding and not neutralization. Even against viruses that had escaped neutralization, ADCC reporter activity was still present as long as binding was maintained. These data are important to consider when determining if MAbs can still protect against a given isolate that may have escaped neutralization. Binding and ADCC activity are more critical for protection compared to neutralization in the case of these MAbs.

All viruses, including the irrelevant IgG control, acquired mutations R62K, D239G, and R240Q in the HA head domain. The mutation R62K is located further down on the HA, near the stalk domain, and may be involved in HA stability. It was observed in viral isolates circulating in 2016, but its effect has not yet been characterized (43). Both D239 and R240 are located near the receptor binding domain, and mutations at these sites have been reported to increase virus growth in eggs and MDCK cells (20, 44). The mutations are most likely cell culture adaptations and could explain why stalk EMVs did not have fitness losses in cell culture growth curves. In addition, the KB2 H45R EMV had an A152S mutation, which has also been implicated as a cell culture adaptation (20). Finally, the irrelevant IgG control virus had an additional HA mutation at residue K163. This residue is located within the antigenic site Sa and has been implicated in antigenic drift because of its variability over time (45). Based on the reports for each HA head mutation in the literature and, since almost all were present in the control virus, we concluded that they were an unlikely cause of the stalk escape phenotypes observed.

The stalk mutation A388T was found in four of six of the EMVs, and an escape mutation at the same position (A388V) has previously been reported during the original characterization of MAb 6F12 (30). It is the only stalk mutation that we found that is located on the interior of the HA, meaning that it is not readily accessible to antibodies in the HA prefusion conformation. A388T may be involved in escape from stalk MAbs by altering the conformation of the antibody binding site or by impacting on the fusion process. It seems to be the critical residue for MAb 6F12 since all EMVs have escaped its neutralization. However, the 6F12 A388T EMV is still neutralized by the broadest MAbs in our panel, indicating that multiple mutations are necessary to escape from these MAbs.

All EMVs were attenuated *in vivo* compared to the irrelevant IgG control virus. Viruses with two or more stalk mutations were no longer lethal, whereas those with a single mutation had 10- to 100-fold increases in their mLD_50_ values. From these data, we concluded that whereas the viruses may have adapted to cell culture as described above, their stalk mutations are impactful enough to lead to attenuation *in vivo*. Attenuation indicates that the acquired HA stalk mutations reduce virus fitness and may be the reason why the identified mutations do not readily appear in nature. This conclusion is bolstered by the irrelevant IgG control virus, which has no HA stalk mutations but shared HA head mutations with the EMVs, yet had a low mLD_50_ of 7 PFU. Viruses that were not completely attenuated were used to challenge mice that had been vaccinated using the cHA approach. All vaccinated mice were fully protected from the EMVs and lost 5 to 10% of their body weights. Vaccinated mice were naive toward the A/Netherlands/602/2009 head domain, implying that protection was driven by stalk-specific antibody responses upon vaccination. We further confirmed this through microneutralization assays. We found that prechallenge sera of mice had negligible neutralizing activity compared to a positive-control MAb (data not shown). The lack of strong neutralization—as seen for other stalk-based vaccines in animal models as well (46, 47)—indicates that other factors, such as Fc-FcR based effector functions, are part of the mechanism for protection. This challenge study reinforces the growing evidence that stalk-based vaccines can protect from viruses that have mutations in their HA, leading to a more “universal” approach in vaccine design that is difficult to escape.

Antigenic drift in the HA head domain is generally caused by focused antibody responses against specific residues in major antigenic sites. As of now, the HA stalk domain has not been directly targeted by focused antibody responses. As a result, the HA stalk does not readily undergo antigenic drift. It could be assumed that widespread use of stalk-based vaccines would begin to generate a polyclonal response focused on the HA stalk domain in the population. Our work indicates that even under direct selection pressures, the HA stalk domain does not mutate easily (mutations began to appear after 8 to 15 passages). No stalk escape mutations led to complete escape from all three MAb properties tested here: neutralization, binding, and ADCC activity. Together, these data suggest that while antibody-related antigenic drift in the HA stalk domain is possible, it is not enough to evade a polyclonal antibody response generated by vaccination. Eventually, an accumulation of many mutations may produce viruses that have drifted HA stalk domains. However, drifted viruses will have an uphill battle because they need to escape broadly reactive MAbs while maintaining fitness. These results reinforce the growing body of evidence that stalk based vaccines can remain protective against antigenically distinct viruses over time.

## MATERIALS AND METHODS

### Cells and viruses.

MDCK cells (ATCC CCL-34) and A549 cells (ATCC CCL-185) were obtained from the American Type Culture Collection. Cells were propagated in Dulbecco's modified Eagle's medium (cDMEM; 1× DMEM [Gibco] supplemented with 10% heat-inactivated fetal bovine serum [Sigma-Aldrich], 1% 100 U/ml penicillin–100 μg/ml streptomycin solution [Gibco], and 1% 1 M 4-(2-hydroxyethyl)-1-piperazineethanesulfonic acid [HEPES, Gibco]) and then incubated at 37°C with 5% CO_2_. A/Netherlands/602/2009 (pdmH1N1) was grown in our laboratory using 10-day-old specific-pathogen-free (SPF) embryonated chicken eggs (Charles River Laboratories) at 37°C for 2 days (48).

### MAb production.

Murine MAbs 6F12 and KB2 were originally produced from hybridoma fusions (30, 32). We expanded hybridomas using methods described previously (30, 32). Briefly, frozen hybridoma stocks were thawed at room temperature and spun at 200 × *g* for 5 min. Next, they were resuspended in 2 ml of ClonaCell-HY growth medium E (Stem Cell Technologies), transferred to a 6-well plate (Corning), and incubated for 1 to 2 days at 37°C and 5% CO_2_. The cells were then expanded into a T-25 flask (Falcon) in medium E and incubated for another 1 to 2 days. The cells were expanded to a T-75 flask (Falcon) using a 1:1 ratio of medium containing medium E and Hybridoma SFM (Thermo Fisher). The cells were further expanded using Hybridoma SFM medium until the desired amount of MAb was produced. Human MAbs 2B06, 2G02, FI6, and CR9114 were produced using transient transfections in Expi293F cells (Thermo Fisher) propagated in Expi293 expression medium (Thermo Fisher) at 37°C with 8% CO_2_ while shaking. Plasmids were obtained from the laboratory of Patrick Wilson. Transient transfections were performed using an ExpiFectamine 293 transfection kit (Thermo Fisher) according to the manufacturer’s instructions. All MAbs were purified using gravity flow with protein G-Sepharose-packed columns as described previously (30). MAbs were eluted into a 50-ml Falcon tube with 5 ml of 2 M Tris (pH 10) and then concentrated using Amicon Ultra 30-kDa centrifugal filter units (Millipore).

### Escape mutant virus generation.

Stalk MAb EMVs were produced by serial passaging in MDCK cells. First, virus was diluted to an MOI of 0.01 (1 × 10^3^ PFU in a 12-well plate [Corning]) in 1× minimal essential medium (MEM; 10% 10× MEM [Gibco], 2 mM l-glutamine [Gibco], 0.1% sodium bicarbonate [Gibco], 10 mM HEPES, 1% 100 U/ml penicillin–100 μg/ml streptomycin solution, and 0.2% BSA) supplemented with 1 μg/ml tolylsulfonyl phenylalanyl chloromethyl ketone (TPCK)-treated trypsin. Next, 0.25× IC_50_ of MAb was added, and the mixture was incubated for 1 h at room temperature while shaking. The virus-MAb mixture was applied to a confluent monolayer of MDCK cells and incubated at 37°C and 5% CO_2_ for 2 days. The supernatant was collected, and a 1:10 dilution was incubated with 0.5× IC_50_ of MAb as described above. This was continued for up to 10 passages, to 128× IC_50_, with the exception of the CR9114 L335V/D363G/A388T EMV. This EMV was passaged like the others until 8× IC_50_ (6 passages). Then, the MAb concentration would be kept constant for 3 passages before doubling, to a max of 64× IC_50_. Each passage was screened for escape from MAb neutralization by using a plaque assay with 128× IC_50_ of each MAb in the overlay. Plaques were chosen from the first passage where escape was observed and then expanded in 10-day-old SPF eggs at 37°C for 2 days.

### Plaque assay.

We determined virus titers using a standard influenza virus plaque assay. MDCK cells were seeded into sterile 12-well plates (Corning) at 8 × 10^5^ cells/ml in cDMEM at ∼18 h prior to infection. Virus or cell culture supernatant was serially diluted 1:10 in 1× MEM six times. MDCK cells were washed one time with 1× phosphate-buffered saline (PBS) before applying 200 μl of diluted virus. Plates were incubated for a total of 40 min (with shaking every 10 min) at 37°C with 5% CO_2_. The virus was then aspirated, and the cells were covered with an overlay of 2× MEM containing 0.1% diethylaminoethyl (DEAE)-dextran, 1 μg/ml TPCK-treated trypsin, and 0.64% Oxoid agarose. The plates were then incubated at 37°C with 5% CO_2_ for 2 to 3 days (until plaques were visible) and fixed with 3.7% paraformaldehyde (PFA; 37% PFA diluted 1:10 in 1× PBS) overnight at 4°C. Finally, the plates were washed in water to remove the overlay, and then the cells were stained with a solution of 20% methanol containing 0.5% crystal violet powder.

### Immunofluorescence.

MDCK cells were seeded at 2 × 10^4^ cells/ml into a sterile 96-well plate (Corning) and incubated overnight at 37°C with 5% CO_2_. The cells were first washed one time with 1× PBS, and then the virus diluted to an MOI of 3 (1.5 × 10^5^ PFU/well) in 1× MEM was added at 100 μl/well. The plates were incubated overnight at 37°C with 5% CO_2_ and then fixed with 200 μl of 3.7% PFA as described above. The next day, the plates were blocked with 1× PBS containing 3% milk powder (American Bio) and incubated for 1 h at room temperature. Blocking solution was replaced with 100 μl of MAb diluted to 30 μg/well in 1% milk. The plates were incubated for 1 h at room temperature while shaking. The cells were then washed three times using 1× PBS, and secondary antibody Alexa Fluor 488-goat anti-mouse IgG(H+L) (Invitrogen) or Alexa Fluor 488-goat anti-human IgG(H+L) (Invitrogen) was added in 1% milk at 100 μl/well. The plates were incubated in the dark at room temperature for 1 h with shaking. Secondary antibodies were removed, and the cells were washed again three times with 1× PBS. A final 50 μl of 1× PBS was added to each well to prevent the monolayer from drying out. The immunofluorescence was measured and visualized using a Celigo S adherent cell cytometer (Nexcelom Bioscience) with the two-channel target 1+2 (merge) setting. Exposure time, gain, and focus (set on a single channel using image-based autofocus with the 488-nm signal as the target) were automatically determined by the machine. The total fluorescence intensity was calculated by using the default analysis settings and downloaded for the percent fluorescence calculations.

### Plaque reduction neutralization assay.

MDCK cells were seeded onto 12-well plates as described above. The next day, MAb was serially diluted 1:5, starting from 100 μg/ml, six times in 1× MEM. Virus was added to MAb dilutions at ∼30 PFU/well (50 μl of 1.5 × 10^3^ PFU/ml), followed by incubation for 1 h at room temperature while shaking. The cells were infected as described in the plaque assay protocol above. In the meantime, the overlay was made by first serially diluting MAbs 1:5, starting from 100 μg/ml, six times in 2× MEM. A mixture of 0.1% 1× DEAE-dextrane and 0.001% TPCK-treated trypsin was diluted in sterile water and added at a 1:3 ratio to the diluted MAbs. Immediately after aspirating the inoculum, 2% Oxoid agarose heated to 56°C was added to each MAb dilution at a 1:2 ratio to a total volume of 1 ml. Each inoculum MAb dilution was covered with an overlay with a matching MAb concentration. The plates were then incubated for 2 days at 37°C with 5% CO_2_ and fixed/stained as described above.

### Antibody-dependent cell-mediated cytotoxicity assay.

ADCC assays were performed using a Promega ADCC reporter assay kit with either mFcγRIV (mouse) or FcγRIIIa (human) effector cells according to the manufacturer’s instructions (41, 49). Briefly, MDCK cells were seeded in white-bottom, sterile 96-well plates at 3 × 10^4^ cells/well in cDMEM, followed by incubation overnight at 37°C with 5% CO_2_. The next day, the cells were washed once with 1× PBS and infected with virus diluted to an MOI of 3 (1.5 × 10^5^ PFU/well) in 100 μl of 1× MEM. The plates were again incubated overnight at 37°C with 5% CO_2_. MAbs were diluted to 30 μg/ml in in Roswell Park Memorial Institute (RPMI) 1640 medium and then diluted 1:2 across the plate (12 times). The inoculum was carefully aspirated so that the monolayer was not disturbed, and 25 μl of RPMI 1640 was added to each well. Next, 50 μl of each MAb dilution was added to each well. The ADCC effector cells were then diluted to 75,000 cells/well in 25 μl of RPMI 1640 media and added to the plate, which was incubated for 6 h at 37°C with 5% CO_2_. The ADCC kit luciferase assay reagent was added to each well at a volume of 75 μl and immediately read using the luminescence setting on a Synergy H1 hybrid multimode microplate reader (BioTek).

### RNA extractions and deep sequencing.

RNA was extracted from virus containing allantoic fluid using an E.Z.N.A. viral RNA extraction kit (Omega Bio-Tek) according to the manufacturer’s instructions and stored at −80°C for future use. Next generation sequencing was performed using a MiSeq v2, 300 cycle reagent kit (Illumina). A customized pipeline that has been implemented at the Icahn School of Medicine at Mount Sinai was used for genome assembly (50). The assembled segments were aligned using MUSCLE in MEGA 7.0 (51) to the wild-type pdmH1N1 deep-sequenced virus used at the beginning of the escape passaging. Of note, the pdmH1N1 virus used in this study has two HA mutations, N173D and Q240R, compared to the sequence that can be found on the Global Initiative on Sharing All Influenza Data (GISAID) database. The CR9114 L335V/D363G/A388T EMV HA mutations were found using Sanger sequencing through Genewiz.

### Phylogenetic tree and percent conservation.

The phylogenetic tree was built using 18 representative HA protein sequences from each influenza A virus HA subtype (H1, A/California/04/2009; H2, A/mallard/Southcentral Alaska/12ML01615/2014; H3, A/Florida/02/2017; H4, A/swine/Missouri/A01727926/2015; H5, A/mallard/Alaska/AH0088535/2016; H6, A/duck/Ganzhou/GZ151/2016; H7, A/chicken/Puebla/CPA-03309-16-CENASA-95076/2016; H8, A/mallard/Interior Alaska/12ML00058/2012; H9, A/chicken/Ganzhou/GZ126/2016; H10, A/duck/Mongolia/709/2015; H11, A/mallard/California/UCD1154/2015; H12, A/guinea fowl/Massachusetts/14075-3/2013; H13, A/glaucous-winged gull/Southcentral Alaska/15MB02016/2015; H14, A/blue-winged teal/Texas/UGAI15-6890/2015; H15, A/mallard/Novomychalivka/2-23-12/2010; H16, A/glaucous-winged gull/Southcentral Alaska/15MB01758/2015; H17, A/yellow shouldered bat/Guatemala/060/2010; H18, A/flat-faced bat/Peru/033/2010) and 3 representative influenza B viruses (B/Lee/1940, B/Victoria/1/2014, and B/Yamagata/16/1988). Sequences were obtained from GISAID and aligned with the MUSCLE algorithm within MEGA 7.0 (51). A maximum-likelihood tree was constructed using RAxML and visualized through FigTree (52). Conservation of each mutated stalk residue was determined using 2,905 sequences spanning from 1918 to 2018 obtained from GISAID and aligned using the MUSCLE algorithm within MEGA 7.0 (51). The data set was then uploaded to the Shannon Entropy-One webserver (https://www.hiv.lanl.gov/) to determine site-by-site variation.

### Growth kinetics.

MDCK and A549 cells were seeded in 24-well plates at a density of 8 × 10^5^ cells/ml, with 500 μl per well, and incubated overnight. The cells were then infected with EMVs at an MOI of 0.01 (5 × 10^3^ PFU/well) in 1× MEM containing 0.0004% TPCK-treated trypsin and incubated for 60 h at 37°C with 5% CO_2_. An aliquot of the original inoculum was immediately collected and treated as T0 for the experiments. Cell culture supernatant was collected every 12 h and frozen at −20°C for further use. Virus titers were determined using plaque assays (in biological duplicates).

### Animal experiments.

Animal experiments were performed in accordance with protocols approved by the Institutional Animal Care and Use Committee at the Icahn School of Medicine at Mount Sinai. All experiments were conducted using female mice at an age of 6 to 8 weeks (species Mus musculus, strain BALB/c) from The Jackson Laboratory.

For assessing the stability of escape mutations *in vivo*, mice were infected with 1 × 10^4^ PFU of each EMV (*n* = 3), and their lungs were collected at 3 days postinfection. The lungs were homogenized, and a 1:100 dilution of the lung homogenate was injected into 10-day-old SPF eggs. The eggs were incubated for 2 days at 37°C. Allantoic fluid was collected and viral RNA was extracted. Reverse transcription-PCR products were sent to Genewiz for Sanger sequencing.

Mouse LD_50_ experiments were conducted by infecting three randomly selected mice intranasally with each virus dilution. Challenge experiments used five randomly selected mice per group. Each challenge virus had two groups: a vaccinated group and a negative-control group. Vaccinated mice received a prime of 1 × 10^4^ PFU of A/Yamagata/16/1988 virus containing a cH9/1 HA (22, 26). Three weeks later, they were boosted with 10 μg of cH8/1 protein adjuvanted with 10 μg of polyI:C in 1× PBS at 100 μl per mouse. Mice were given 50 μl of protein intranasally and 50 μl intramuscularly according to our initial protocol (22). After an additional 3 weeks, mice were boosted again with cH5/1 protein adjuvanted with polyI:C. The cHA constructs contained an A/California/04/2009 (pdmH1N1) stalk domain with a stabilizing mutation to ensure proper conformation (27, 53). Negative-control mice followed the same vaccination timeline. However, these mice were primed with wild-type B/Yamagata/16/1988 and boosted with 10 μg of BSA adjuvanted with 10 μg of polyI:C. At 4 weeks after the final boost, the mice were intranasally challenged with 20× mLD_50_ of each EMV. For all mouse experiments, body weight and survival were monitored for 14 days postinfection. Any mice that lost more than 25% of their initial body weight before the end of the 14 days were euthanized.

### Calculations and data analysis.

IC_50_ calculations were done using a nonlinear regression (four parameters) based on log_10_-transformed antibody concentrations in Prism 7.0. AUC values were calculated using the area-under-the-curve function in Prism. All graphs were visualized using Prism.
