# Supplementary figures and images for: Mutations in the Hemagglutinin Stalk Domain Do Not Permit Escape from a Protective, Stalk-Based Vaccine-Induced Immune Response in the Mouse Model
Source: mBio. 2021 Feb 16;12(1):e03617-20. doi: 10.1128/mBio.03617-20 (PMC8545130; doi:10.1128/mBio.03617-20)

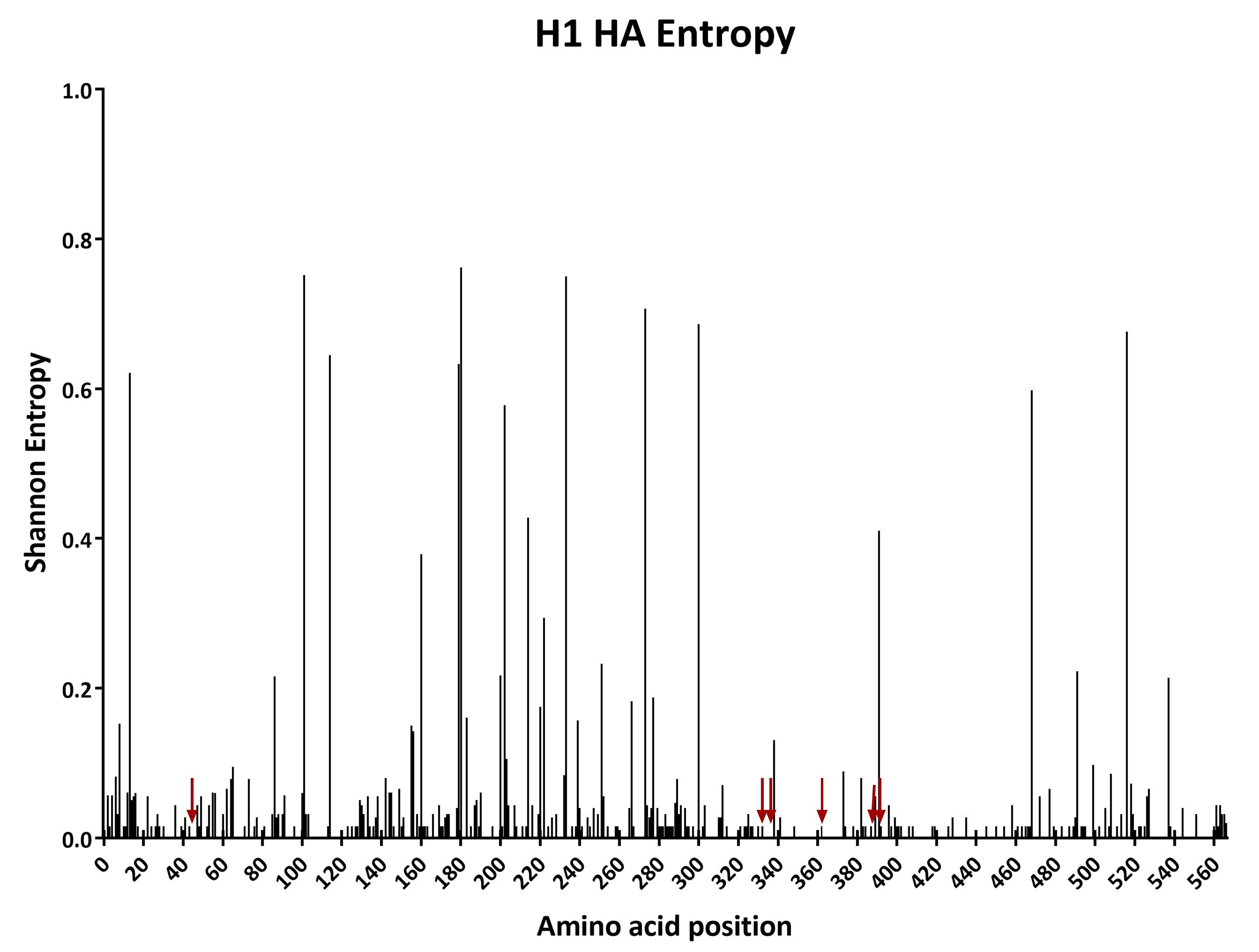

Supplement: FIG S1 [file mbio.03617-20-sf001.tif]
